# The causal web of foetal alcohol spectrum disorders: a review and causal diagram

**DOI:** 10.1007/s00787-018-1264-3

**Published:** 2019-01-16

**Authors:** Cheryl McQuire, R. Daniel, L. Hurt, A. Kemp, S. Paranjothy

**Affiliations:** 1 Population Health Sciences, Bristol Medical School, University of Bristol, Canynge Hall, 39 Whatley Road, Bristol, BS8 2PS UK; 2 Division of Population Medicine, Cardiff University, 3rd Floor, Neuadd Meirionnydd, Heath Park, Cardiff, CF14 4YS UK

**Keywords:** Foetal alcohol spectrum disorders, Causal diagram, Directed acyclic graph, Causal inference, Review, Prenatal exposures

## Abstract

Foetal alcohol spectrum disorders (FASDs) are a leading cause of developmental disability. Prenatal alcohol use is the sole necessary cause of FASD, but it is not always sufficient. Multiple factors influence a child’s susceptibility to FASD following prenatal alcohol exposure. Much of the FASD risk factor literature has been limited to discussions of association, rather than causation. While knowledge of predictor variables is important for identifying who is most at risk of FASD and for targeting interventions, causal knowledge is important for identifying effective mechanisms for prevention and intervention programmes. We conducted a systematic search and narrative synthesis of the evidence and used this to create a causal diagram (directed acyclic graph; DAG) to describe the causal pathways to FASD. Our results show that the aetiology of FASD is multifaceted and complex. FASD risk is determined by a range of lifestyle, sociodemographic, maternal, social, gestational, and genetic factors. The causal diagram that we present in this review provides a comprehensive summary of causal risk factors for FASD and can be used as a tool to inform data collection and statistical modelling strategies to minimise bias in future studies of FASD.

**Electronic supplementary material:**

The online version of this article (10.1007/s00787-018-1264-3) contains supplementary material, which is available to authorized users.

## Introduction

Foetal alcohol spectrum disorder (FASD) is an umbrella term that is used to describe a range of lifelong disabilities caused by prenatal alcohol exposure [1]. Individuals with FASD have neurodevelopmental impairments and some will also have growth deficiency and a distinctive facial phenotype, characterised by a thin upper lip, smooth philtrum and short palpebral fissure length [2]. Conservative estimates suggest that around 2–5% of children in the general population of Europe and North America have FASD, making it one of the leading causes of preventable developmental disability worldwide [3, 4]. Maternal alcohol use is the sole necessary cause of FASD, but it is not always sufficient [5]. Among women who drink any amount of alcohol in pregnancy, an estimated one in 13 will have a child with FASD and one in 67 will have a child with foetal alcohol syndrome (FAS) [4, 6]. Alcohol interacts with multiple factors in a complex process to determine offspring outcome [7]. Accordingly, rather than a simple causal chain, the image of a spider’s web has been considered most appropriate for describing the causal context of FASD [8].

Causal inference, the science of inferring the presence and magnitude of cause–effect relationships from data, is a central aim of epidemiology [9, 10]. However, much of the existing FASD literature simply lists risk factors and reports associations with little consideration of the underlying causal structure. The term ‘risk factor’ obscures the distinction between a predictor variable and a cause [11]. While knowledge of predictor variables is important for identifying who is most at risk of FASD and for targeting interventions, causal knowledge is important for identifying effective mechanisms for prevention and intervention programmes [12].

Randomised controlled trials can be used to estimate causal effects. However, for many public health issues, including prenatal alcohol exposure (PAE), randomisation of exposure is unethical and/or unfeasible and, therefore, it is necessary to rely on observational data. Measures of (conditional) association from observational designs can be used to estimate causal effects if conditional exchangeability is created by appropriate control of bias, such as adjustment for confounders [11]. Causal diagrams, known as directed acyclic graphs (DAGs), are gaining popularity as a gold standard method for supporting causal inference and reducing bias in epidemiological studies [13]. Judea Pearl devised the unifying framework that provided this graphical method and formal language for causal inference [14]. Pearl reasoned that the combination of observed data plus causal knowledge allows individuals to move beyond the realm of statistical association to that of causality. DAGs provide a tool for explicitly characterising assumptions about the causal relationships between exposures, covariates, and outcomes [15]. The graphical rules that underpin the interpretation of causal DAGs have a well-established mathematical basis [14]. They provide a systematic method for identifying which variables should be controlled for in the analysis, and which should not, to minimise bias in effect estimates, on the basis of the assumptions encoded in the DAG [14]. In scenarios where DAGs contain a large number of variables they become less clear as visual overviews of the causal context, but maintain their utility as a theory driven approach to statistical modelling strategies. In contrast to data-driven approaches, it is the hypothesised causal relationships depicted by the DAG, rather than measures of statistical significance that can be used to guide variable selection in subsequent quantitative analyses [16]. A detailed description of DAG language and theory is provided in Online Resource 1. In this review, we conduct a narrative literature synthesis and present a DAG to describe the hypothesised causal structure of the variables that are involved in the pathways to FASD. We also provide a worked example of how the DAG can be used to support statistical modelling strategies.

## Method

### Literature search

We searched Medline from inception to 2nd March 2016 for existing systematic reviews of FASD risk factors using combinations of medical search headings (MeSH) and free text search terms for FASD, risk factors and reviews, as described in Online Resource 2. This search produced two references, one of which, by Esper et al. [17], was relevant to the aims of this review. To obtain more information about possible causal structures, we searched the full text articles of the included studies from this review and conducted separate searches for each risk factor in Medline. In each search, we combined search terms for FASD and the relevant risk factor (see Online Resource 2 for example search on FASD and stress). Supplementary sources were searched to identify any risk factors that were not identified in the initial review and to provide further information on identified risk factors. Sources included: the Infant Feeding Survey [18–20], publications from the Millennium Cohort Study [21] and ALSPAC [22], the National Organization on Fetal Alcohol Syndrome and EUFASD newsletters, a search of study reference lists, Research Gate and FASD conference abstracts [23–25]. Supplementary searches were concluded on the 22nd December 2017.

### Evidence synthesis

To determine the most plausible structure for the aetiology of FASD, we used informal triangulation methods to evaluate the evidence from the literature search. Unlike systematic reviews, which seek to compare results from studies that are relatively homogenous, triangulation seeks to compare evidence from studies from a diverse range of approaches. The advantage of triangulation is that any biases are assumed to differ across the diverse study types. This increases confidence in causal inferences if similar effect estimates are obtained [26].

### DAG construction

We used the DAGitty platform [27] to draw and interpret a DAG based on the evidence synthesis. DAGitty code is provided in Online Resource 3.

## Results

**Fig. 1:**
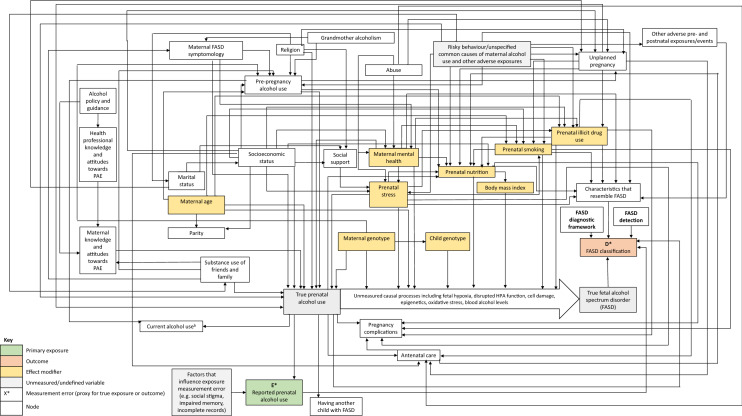
Directed acyclic graph (DAG) depicting the hypothesised causal pathways to FASD. Note: evidence of prenatal alcohol exposure is required to consider a diagnosis of FASD. **a** Rural residence may be Rural residence may be a risk factor that is particular to farming communities in South Africa, due to normative binge pattern drinking behaviour and adverse social conditions, **b** Having another child with FASD and current alcohol use are descendants of prenatal alcohol use and descendants of all factors that influence alcohol use before and during pregnancy. These connecting arcs have not been depicted in the DAG for clarity of presentation

The DAG (Fig. 1) provides a visual description of the hypothesised aetiological context of FASD, based on the evidence synthesis.

In this section, we present the narrative synthesis of evidence that supports the inclusion of factors in the DAG, and their position, followed by a worked example of how the DAG can be used to inform statistical modelling strategies. In Online Resource 4, we provide a more detailed description of the groupings of variables that were derived following the evidence synthesis, to enable further comparison of the literature review with the causal (and non-causal) relationships depicted in the DAG.

### Patterns of maternal alcohol use (prenatal alcohol exposure)

During pregnancy, alcohol consumed by the mother passes freely through the placenta and within 1 h the level of alcohol within the foetal bloodstream approximates that of the mother [28]. For simplicity, we use term prenatal alcohol exposure (PAE) to refer to maternal alcohol use, although it is important to note that maternal alcohol use is just one element of foetal alcohol exposure, which is also influenced by maternal and infant metabolism and other modifying factors, described in this review.

The impact of PAE varies according to the dose, frequency and timing of exposure. However, residual confounding, measurement error and individual variability across a vast range of covariates complicate efforts to determine what pattern of maternal alcohol use will lead to FASD in an individual case. Nevertheless, on average, binge and heavy chronic patterns of maternal alcohol use are most likely to result in FASD [29–34]. Evidence on the effects of low to moderate PAE on developmental outcomes is limited and inconsistent, ranging from evidence of harm, to no effect, to evidence of slight benefit [35–44]. For example, Kelly et al. found that 3-year-old boys who were born to mothers who reported drinking no more than one to two units of alcohol per week or per occasion during pregnancy had a lower risk of hyperactivity and conduct problems than those born to abstainers [40]. Negative control studies that compare the strength of association for maternal and paternal exposures have also been used to investigate the causal effects of low to moderate PAE, but did not find evidence of intrauterine effects on child IQ or head circumference [41, 42]. In contrast, Mendelian randomisation studies have offered evidence that low to moderate PAE can cause persistent conduct problems and adversely affect cognitive and academic outcomes [37, 38, 43]. Despite inconsistencies in the evidence, Mendelian randomisation studies provide stronger causal evidence than traditional observational studies that low to moderate alcohol use can cause adverse developmental outcomes. They suggest that the apparent null or protective effects of low to moderate PAE are likely to be due to residual confounding, owing to the socioeconomic patterning of prenatal alcohol use [36, 37, 44].

### Alcohol use before pregnancy

Mothers of children with FASD are more likely to drink at high levels before pregnancy, to have had longer ‘drinking careers’ (i.e. more years of drinking alcohol), and to have a history of alcohol abuse, relative to controls [30, 32, 33, 45]. Chronic alcohol use impairs the functioning of alcohol metabolising enzymes and can lead to malnutrition due to reduced intake and absorption of key nutrients, thus increasing FASD risk [5, 46–49].

In the UK, up to 91% of women aged 16–45 report drinking alcohol and excessive consumption is common [50, 51]. Few women follow guidance for prenatal alcohol use when planning a pregnancy [52] and alcohol use increases the risk of unplanned pregnancy [53–56]. Therefore, alcohol use prior to pregnancy is a significant risk factor for FASD.

### Postnatal alcohol use

Mothers of children with FASD drink more heavily than controls postnatally [33, 57–65]. Of course, heavy postnatal alcohol use cannot cause FASD among children who have already been born. It can, however, indicate a continuing pattern of heavy alcohol use and serve as a risk marker for previous and future alcohol-exposed pregnancies. Heavy postnatal alcohol consumption may be a useful proxy for PAE. Studies from the USA and Italy found that self-reported prenatal alcohol use was not consistently different between mothers of children with FASD and controls, but current consumption was higher [34, 63, 64]. In contrast, studies in the Western Cape of South Africa (where maternal self-report is believed to be more reliable, due to normative binge drinking patterns and higher levels of PAE) show consistently higher levels of self-reported PAE and current consumption among mothers of children with FASD [33, 57–60, 62]. Therefore, postnatal drinking patterns may be particularly useful as a risk marker for previous PAE and for future FASD risk in populations that are susceptible to underreporting of PAE.

### Smoking during pregnancy

Prenatal cigarette smoking is more common among mothers of children with FASD than those without [17, 58]. Smoking and PAE may interact synergistically to increase the risk of FASD-related outcomes including low birth weight [66]. Proposed biologic mechanisms for the interaction between tobacco and alcohol include common effects on nutrient availability, oxidative stress and vasoconstriction of the placenta and umbilical cord, which can lead to hypoxia and prolonged uterine exposure to ethanol [5, 66, 67]. In addition to its interactive effect with PAE, prenatal smoking is independently associated with reduced foetal growth, low birthweight and cognitive and behavioural impairment [68–75]. Evidence suggests that the impact of smoking on decreased birth weight is up to three times greater than that of PAE [68, 76]. Therefore, prenatal tobacco exposure can lead to characteristics that resemble FASD, even in the absence of alcohol exposure. Prenatal smoking is more common among mothers with an unplanned pregnancy, mothers with other forms of substance use, mothers who report prenatal stress, younger mothers, and mothers of lower socioeconomic status (SES) [19, 69, 73, 77].

### Illicit drug use during pregnancy

Prenatal illicit drug use is more prevalent among mothers of children with FASD [30, 78] and it can lead to similar physical, cognitive and behavioural impairments that are relevant to FASD diagnosis [79, 80]. Cocaine, opiates and amphetamines have been found to increase the risk of intrauterine growth restriction, low birth weight, small head circumference and congenital anomalies [72, 79–81]. However, the effect of illicit substance exposure on birthweight and foetal growth is thought to be less severe and persistent than that of PAE and prenatal smoking [68, 71]. Children with prenatal illicit substance exposure may show catch-up growth [82] and effects are significantly attenuated following adjustment for adverse social factors [83]. Evidence on the effect of prenatal marijuana exposure on growth outcomes has been inconsistent [68, 71, 84]. However, marijuana use may lead to subtle impairments in cognitive and motor skills in childhood and adolescence [85–87]. In a mouse model study, synthetic cannabinoids were found to interact with ethanol to increase the prevalence of ocular defects [88].

Prenatal cocaine exposure may impair attention, speech and language development [89–91]. Opiate exposure can lead to neonatal abstinence syndrome, which is characterised by abnormal arousal and irritability [80, 81]. Prenatal amphetamine exposure has been linked to smaller subcortical volume, externalising behaviour, and cognitive deficits [92–94]. It is unclear whether illicit drugs modify the effects of PAE [83]; however, prenatal illicit drug exposure and PAE share common biological mechanisms of harm including restricted blood flow to the foetus and altered neuroendocrine regulation [79, 95].

Prenatal illicit drug use is associated with a range of social and psychological factors including low socioeconomic status (SES), stress, mental health problems, abuse and low social support [79, 83, 96]. Although maternal drug use may contribute to subsequent adverse social and psychological outcomes, such factors are primarily perceived as preceding factors that increase the likelihood of drug use [83]. Illicit drug use increases the risk of inadequate prenatal care, pregnancy complications, and poor nutrition and is more common among older women and those with an unplanned pregnancy [79, 83, 95, 97].

### Prenatal nutrition

Prenatal nutrition features at several points in the causal context of FASD. Nutrition modifies the impact of PAE on FASD, influences maternal BMI, and is influenced by maternal substance use, maternal mental health, stress, antenatal care, whether pregnancy was planned or not, and SES [5, 98–101]. Greater consumption of processed foods has been found to be associated with heavier alcohol intake and healthier diets with low-to-moderate alcohol intake during pregnancy [102]. These associations may be due to latent factors such as tendencies towards healthy or unhealthy lifestyle behaviours (depicted as the ‘risky behaviour’ node in the DAG). Among children with PAE, lower calorific intake has been found to increase the risk of FASD, while evidence from animal studies suggests that specific nutrients (including vitamin A, docosahexaenoic acid, folate, zinc, choline, vitamin E, selenium, riboflavin, calcium, docosapentaenoic acid, zinc, B-vitamins, iron and protein) may reduce the risk of FASD-relevant outcomes including physical malformations, growth deficiency, behavioural regulation and memory [98, 99, 103–106]. During pregnancy, mothers of children with FASD report a lower intake of key nutrients and report being hungry more often than controls [59, 107]. Deficient nutrient intake does not, however, fully explain increased risk for FASD. Even with equivalent dietary intake, alcohol-exposed rats weigh less and produce offspring with poorer outcomes than unexposed rats [104, 108]. Alcohol competes with nutrients that are essential for foetal development due to shared metabolic pathways [109, 110] and can lead to impaired placental blood flow and nutrient transportation [49, 98, 111, 112]. Nutritional supplementation has been found to attenuate FASD symptomology in some animal models [98]. Results in human studies are limited and inconsistent [113–115].

### Socioeconomic status (SES)

Indicators of SES including low maternal education and income have been identified as risk factors for FASD [17, 58]. Perhaps counterintuitively in light of these results, the UK Infant Feeding Survey has consistently found that women within higher social classes are more likely to drink during pregnancy than those from lower social classes [18, 19, 116, 117]. UK-based cohort studies have echoed these results, showing that women who drink in pregnancy are more highly educated, less likely to live in deprived areas, and more likely to be employed than abstainers [37, 118, 119]. Although high SES is associated with an increased risk of prenatal alcohol use, mothers of low SES who do drink during pregnancy are more likely to do so in a binge pattern [37]. As well as differences in the social patterning of alcohol use, children born to high SES mothers may be relatively protected against the harms of PAE due to factors associated with social advantage [39]. These factors are included in the DAG as consequences of SES and, among others, include differences in social support, stress, prenatal nutrition and prenatal smoking, which could offer potential targets for intervention.

Based on the available evidence, it is hypothesised that SES is associated with FASD via the causal pathways proposed by Abel et al. [5], in which low SES contributes to FASD through its influence on drinking patterns, stress, mental health, illicit substance use, smoking and nutrition.

### Maternal age

Several studies have found that older maternal age is associated with an increased risk of FASD [17, 58]. Older mothers are more likely to drink during pregnancy than younger mothers, although binge drinking is more common among younger mothers [18, 19, 116–118]. Blood alcohol concentration (BAC) per unit dose increases with age, leading to higher levels of exposure per unit consumed among the offspring of older mothers [120–123]. Higher BACs may be partly explained by age-related changes in body composition including an increased body-fat-to-water ratio among older individuals [120, 122–124]. Some studies have also found differences in rates of ethanol metabolism among older participants, although results are mixed [121–123, 125–130]. Chronic alcohol exposure, which is associated with maternal age, can impair nutrient transportation [112, 131]. Thus, maternal age is proposed to influence the risk of FASD through differential patterns of PAE, BAC and effects on nutrient availability.

### Marital status

Mothers of children with FASD are less likely to be married and more likely to be cohabiting with a partner than controls [17]. Existing studies do not offer an explanation as to the causal basis of this relationship; however, it is possible that marriage may protect against FASD by offering a form of social support. It may also indicate fewer relationship problems, which have been cited as a source of prenatal stress and predictor of PAE among mothers of children with FASD. In addition, marriage is associated with higher SES and lower risk of unplanned pregnancy, which predict a lower risk of FASD.

### Religion

Esper et al. concluded that ‘less religious’ women had an increased risk of having a child with FASD [17]. However, evidence is mixed. Three South African studies found that mothers of children with FASD reported a lower frequency of church attendance and prayer than controls [33, 59, 62]. Conversely, studies in Italy found that women categorised as more religious were more likely have a child with FASD than those classed as less religious [64, 65]. Other studies have found no significant differences in the religious practices of mothers of children with FASD and those without [132]. These studies do not provide any insight into possible causal relationships between religion and FASD. However, the extent to which religion may protect against, or be a risk factor for, FASD is likely to depend on its association with more proximal risk factors for FASD, such as alcohol use, unplanned pregnancy and social support. For example, religions differ in their stance towards alcohol consumption. The Islamic faith promotes abstinence from alcohol and abstinence is high among Muslims [133]. The prevalence of FASD is 50 times lower than the global average in the World Health Organisation (WHO) Eastern Mediterranean Region, where the population is predominantly Muslim [6]. In contrast, wine is part of Communion in the Catholic faith and abstinence is much less common among Catholics [133]. Astley et al. found that mothers of children with FAS were more likely to become abstinent in the future if they reported a religious affiliation and more satisfactory support networks [134]. Finer et al. found that religion was associated with a reduced risk of unplanned pregnancy [135]. Therefore, religion may protect against FASD if the faith promotes abstinence from alcohol, or drinking in moderation, reduces the risk of unplanned pregnancy, and offers social support.

### Parity

Parity is higher among mothers of children with FASD, relative to controls [17, 58]. Some authors have suggested that higher parity could increase susceptibility to alcohol teratogenicity due to greater levels of uterine collagen and elastin, which reduce blood flow and contribute to foetal hypoxia [5]. However, experimental studies suggest that it is maternal age, rather than parity that modifies the effect of PAE on offspring outcomes. When rats of the same age, but different parity, are exposed to equivalent levels of alcohol, the number of birth defects is comparable across groups. In contrast, older rats are more likely to produce offspring with birth defects following PAE than younger rats, when parity is equivalent [136, 137].

### Pregnancy complications

Mothers of children with FASD report more complications including preterm delivery, foetal distress, miscarriage, stillbirth and admission to special care baby units [31, 33, 138, 139]. Pre- and perinatal complications are an effect of PAE and may serve as a marker of exposure to factors associated with increased risk of FASD. Complications during pregnancy and delivery have also been linked to other exposures including prenatal smoking, prenatal illicit drug use, poor nutrition and prenatal stress [31, 33, 95, 138–143]. The risk of pregnancy complications may be mitigated by adherence to antenatal care recommendations and monitoring [97, 144].

### Antenatal care

Compared to controls, mothers of children with FASD are less likely to have received antenatal care, more likely to have accessed care late in pregnancy and more likely to have attended fewer antenatal appointments [30, 31, 78, 138, 139]. Women who misuse substances may be likely to access prenatal care due to fear about negative staff attitudes, the involvement of children’s services, and feelings of guilt [97]. As well as influencing uptake of antenatal care, prenatal substance use may be influenced by antenatal care. For example, mothers accessing antenatal care via the National Health Service (NHS) are provided with information about the risks of prenatal substance use and support organisations [145]. In the USA, pregnant women who have health insurance are approximately 50% less likely to have consumed alcohol in the last month, further suggesting that antenatal care can influence PAE (although this relationship may also be due to the association between PAE and SES) [146]. Therefore, there is a time-dependent causal relationship between PAE [and similarly other types of prenatal substance use (e.g. smoking, illicit drug use)] and antenatal care. For example, alcohol use during very early pregnancy (time 1) may affect attendance at the first antenatal booking appointment (at 8–12 weeks in the NHS), which may then affect alcohol use in the second trimester (time 2) and so on. As it is not possible to have feedback loops within DAGs, the full sequence can be conceptualised as a series of time-dependent factors, for example: *substance use before pregnancy → prenatal alcohol use at time 1 → attendance at antenatal care → prenatal alcohol use at time 2.* To avoid over-cluttering the DAG, we depicted antenatal care as a consequence of prenatal substance use (time 1 relationship only).

### Unplanned pregnancy

Worldwide, 40% of pregnancies are unplanned [147], ranging from 16% in the UK [56], 35% in Africa, and 51% in North America [147]. Unplanned pregnancy is a risk factor for FASD. Among a sample of mothers of children with foetal alcohol syndrome (FAS) in the USA, 73% of live births were unplanned [134]. Unplanned pregnancy is more common among women who drink regularly and/or binge drink [53–56] and binge drinking is associated with inadequate methods of contraception [148]. Therefore, the risk of unintended PAE during the periconceptual period is high. Women who have an unplanned pregnancy are less likely than those with a planned pregnancy to follow advice for prenatal lifestyle behaviours including alcohol use, smoking and diet [52, 149, 150]. A qualitative study of pregnant women who attended alcohol establishments in South Africa suggested that some women with unplanned pregnancies continued to drink because they did not feel a connection with their unborn baby, and some women reported drinking in an attempt to abort the foetus [151].

Other factors associated with an increased risk of unplanned pregnancy include low SES, single marital status, exposure to intimate partner violence, smoking, illicit drug use and younger maternal age, while religion is associated with a decreased risk [53, 54, 56, 134, 135, 152]. Some of these factors may be causal and others may be risk markers for unplanned pregnancy due to their association with other causal factors. For example, low SES may have a causal relationship with unplanned pregnancy, as women may be less likely to commit limited financial resources to contraceptives. Studies conducted in the USA show that unplanned pregnancy is more common among poorer women [135] and in one study 43% of women who gave birth to a child with FAS reported that they could not afford contraception [134]. Conversely, socioeconomic status was not found to be associated with unplanned pregnancy in a UK study, where contraception is available for free [56]. Intimate partner violence may be causally associated with unplanned pregnancy due to an increased risk of sexual coercion, and sabotage of contraception [152, 153]. Substance use before pregnancy may be causally associated with unplanned pregnancy, as it increases the likelihood of not using contraception [148, 154]. Further consequences of unplanned pregnancy include late prenatal care and complications during pregnancy [53–55, 135, 155].

### Prenatal stress

Mothers of children with FASD are more likely to report that pregnancy was a particularly stressful time and to have experienced stressful life events, including abuse and interpersonal violence [8, 17]. Qualitative evidence suggests that some mothers may use alcohol to alleviate stress during pregnancy [62, 134, 151]. Prenatal stress has been found to predict co-occurrence of PAE, prenatal smoking, illicit drug use, and poor nutrition [77, 83]. It is important to acknowledge that substance use may also cause prenatal stress; however, in the literature, stress is typically thought to precede substance use [33]. As well as influencing drinking behaviour, prenatal stress may exacerbate the teratogenic effects of alcohol [156]. In a Ukrainian study, prenatal depression and PAE jointly influenced neurodevelopmental outcomes in infants. This effect may have been partially mediated through prenatal stress [16]. Reviews of animal studies have concluded that PAE and stress operate synergistically to impair developmental outcomes [7]. Primate studies have found that concurrent exposure to prenatal stressors exacerbate alcohol-induced behavioural and neurodevelopmental impairments [157, 158]. However, evidence is inconsistent and not all studies have found an interaction between PAE and stress in producing outcomes relevant to FASD symptomology, possibly as a result of small sample size [159–161].

Several biological mechanisms have been proposed to account for observed interactions between PAE and stress in FASD. One hypothesis is that PAE and prenatal stress produce lasting changes in the functioning of the foetal hypothalamic–pituitary–adrenal (HPA) axis, which is involved in the stress response. This effect is heightened in the presence of both PAE and stress, relative to their individual contribution [162–164]. Endocrine dysregulation may contribute to the risk of FASD by influencing behavioural, cognitive and emotional functioning [157, 163]. Dual exposure may also contribute to foetal hypoxia by restricting uterine blood flow and suppressing foetal breathing. Hypoxia promotes cell damage, which may lead to midfacial abnormalities [156] and can be particularly damaging in areas such as the hippocampus and cerebellum which are involved in attention, motor function and learning [5, 157]. Prenatal stress has been independently linked to adverse child outcomes and congenital anomalies and, therefore, may produce symptoms that resemble FASD [156, 165–168].

### Maternal mental health

Mental health disorders are common among mothers of children with FASD [17, 32, 134]. Among a clinic-based sample of 80 mothers of children with FAS in the USA, 96% had one or more mental health disorders [134]. The mechanisms that link prenatal mental health to child outcomes are poorly understood; however, effects are thought to be facilitated primarily via increased stress hormone circulation and altered HPA functioning, as described in the previous section [169–171]. Bandoli et al. found that unmedicated maternal depression and PAE interacted to predict poorer outcomes on the Bayley Scales of Infant Development (BSID) at ages 6 and 12 months, while maternal depression, alone, was not independently associated with neurodevelopmental outcomes [16]. Santucci et al. also failed to find an association between prenatal depression and BSID scores [172]. Other studies have found an independent association between prenatal depression symptoms and poorer emotional–behavioural and cognitive outcomes in children up to age eight [100, 101]. These effects were thought to be partially mediated through an unhealthy prenatal diet. Overall, results are inconsistent with regard to the independent influence of prenatal mental health on FASD-related symptomology. Following a review of the evidence, Waters et al. concluded that, with the exception of conduct problems, prenatal depression does not appear to independently influence child neurodevelopmental outcomes [170]. Therefore, associations between prenatal mental health and FASD are thought to be largely mediated by the prenatal stress response rather than mental health itself.

In addition to its interaction with PAE, maternal mental health may influence drinking behaviour due to the use of alcohol for self-medication [151, 173], as depression often precedes alcohol use disorder [174]. Mothers who have a child with FAS and subsequently receive mental health treatment are more likely to report abstinence, suggesting a promising target for FASD prevention [134]. Maternal mental health issues may also affect foetal development by increasing the risk of other adverse exposures including illicit substance use, smoking and poor prenatal nutrition [83, 100, 101, 175].

### Social support in pregnancy

Social support during pregnancy is associated with reduced alcohol intake in European and American samples [176, 177]. Social support may protect against PAE by attenuating the impact of stressors and reducing the likelihood that alcohol will be used as a coping strategy. Mothers of children with FASD are more likely to achieve abstinence in the future if they report having a large, satisfactory support network [134]. However, the beneficial effects of social support on alcohol use may be less apparent among women in poverty and within subcultures that normalise drinking in pregnancy [151, 178].

### Abuse

FASD is more common among children born to women who have experienced childhood physical or sexual abuse and/or intimate partner violence [17, 32, 33, 134]. Substances including alcohol may be used as a self-medication strategy to alleviate stress and mental health symptoms among those who have experienced abuse [179]. Abuse influences whether mothers reduce their alcohol intake following pregnancy recognition. 72% of mothers of children with FAS reported that they did not want to reduce their alcohol use because they were in an abusive relationship [134]. Abuse may also influence risk of FASD by acting as a barrier to adequate antenatal care. Women experiencing domestic abuse may be prevented from accessing antenatal care or may be reluctant to access care due to concerns that disclosure of abuse would make the situation worse, or lead to involvement of child protection services [97].

### Maternal physical characteristics

Lower maternal height, weight and BMI have been found to consistently predict FASD in studies in South Africa [33, 58–61, 132]. Studies conducted in Italy and the USA have been less consistent with regard to BMI, but have found that mothers of children with FASD tend to be shorter or weigh less than those without FASD [34, 63, 78, 99]. Maternal physical characteristics influence the distribution of alcohol after it is consumed. As alcohol is distributed in body water, blood alcohol concentrations (BACs) tend to be higher in smaller women [180, 181]. As well as influencing BACs, higher maternal weight and BMI may indicate adequate nutrition. Although higher BMI may protect against FASD, it is important to note that maternal obesity is associated with a range of adverse outcomes including gestational diabetes mellitus, induction of labour and preterm delivery [182].

### Maternal FASD symptomology

Mothers of children with FASD are more likely to have symptoms of FASD themselves, including cognitive impairment and small head circumference [32, 58, 59]. Up to 94% of individuals with FASD have comorbid mental health disorders [183], including conduct disorder (91%), ADHD (51%), and depression (35%) [184]. In turn, impaired mental health increases the risk of PAE. 55% of individuals with FASD experience drug or alcohol dependence later in life [184], thus increasing the risk of FASD in subsequent generations.

### Knowledge and attitudes towards PAE and FASD

Public awareness of the risks of PAE is low. Inconsistencies in guidance and mixed advice from health professionals are likely to contribute this lack of awareness [185]. A UK study, published in 2015, found that 40% of participants did not know the government guidance on PAE. Most participants (71%) also said that government guidance on PAE was unclear and some said that this lack of clarity was likely to lead to messages being disregarded [186]. Health professionals have been found to give mixed advice to women about PAE. The UK Infant Feeding Survey 2010 reported that 30% of expectant mothers were not given any information about PAE. Among women who received advice, 36% were given information on reducing their intake and 29% on stopping drinking [19]. In the absence of clear evidence about a ‘safe’ threshold for PAE, opinions can be polarised. Some groups endorse a ‘no alcohol no risk’ message, while others warn against the ‘policing of pregnancy’ and suggest that women should be free to make an educated choice about whether they drink when trying to conceive and during pregnancy [187, 188]. Behaviour and attitudes towards PAE differ according to social norms and may be influenced by national public health policy [189].

### Alcohol use of friends and family

Mothers of children with FASD report higher levels of alcohol consumption by their partner, family and friends [17, 34]. Ceccanti et al. found that alcohol problems in the family increased the likelihood of having a child with FASD by nine times [63]. Alcohol use by friends and family may impact on PAE in various ways. First, the drinking behaviour of those in close social networks may represent the norms within that context. For example, a binge pattern of drinking may be viewed as less problematic in some communities in South Africa where heavy episodic alcohol use is commonplace [132]. Second, some women may feel coerced into drinking before and during pregnancy as a result of the behaviour of friends and family. In a study of mothers of children with FAS, Astley et al. reported that 36% of women said that they did not want to reduce their prenatal alcohol intake because their partner did not want them to, and 20% because their family and friends did not want them to [134]. Strong correlations between maternal substance use and the substance use of friends and family could also be due to processes of self-selection, in which individuals pick companions who are similar to themselves and who support their behaviour [190]. In the DAG, we represented this mechanism via the unspecified ‘risky behaviour’ node that influences both maternal substance use and choice of social network. Finally, as described above, mothers of children with FASD are more likely to have symptoms of FASD themselves and to report that the maternal grandmother has a history of alcohol problems [32, 59]. This raises the possibility of intergenerational transmission of FASD, a phenomenon that has received support via controlled animal studies [191]. Heavy alcohol use by the maternal grandmother could indicate the presence of a risk genotype for heavy alcohol use [192], could result in epigenetic changes that increase the risk of heavy alcohol use in later generations [191] and could provide a model of problematic alcohol use that is adopted by offspring via social learning [193]. Recent evidence from animal studies suggests that paternal alcohol consumption could influence the epigenetics of sperm DNA by influencing methylation patterns in sites that are important to developmental outcomes [194]. Paternal alcohol use has been associated with FASD symptomology including low birth weight, reduced brain size, microcephaly, impaired learning and hyperresponsiveness [42, 195], although results have been inconsistent [196].

### Having another child with FASD

An early study, published in 1979, found that 70% of children with FAS had a sibling with confirmed or suspected FAS [197]. In the FAS Surveillance Network, between 9 and 29% of mothers have another child with FASD [30]. Having another child with FASD is also a strong predictor of subsequent alcohol-exposed pregnancies [30, 31, 197, 198]. In a cross-sectional study of mothers of children with FAS, 80% subsequently had an unplanned pregnancy, of which 75% were alcohol exposed [134]. In the absence of intervention, women who are at risk of having another child with FASD may exist within the same causal context as that which surrounded their first affected pregnancy. Thus, identifying mothers who have had a child with FASD is an important focus for targeted prevention [198].

### Genetics

Genetic factors modify susceptibility to alcohol-related harm [192, 199–201]. Dizygotic twins have differential susceptibility to FASD, while monozygotic twins have high levels of concordance [202–205], different strains of mice have diverse outcomes according to their genotype [206, 207] and particular genetic variants are more common among alcohol-exposed children who develop FASD, compared to those who do not [208, 209]. A range of genes have been investigated in animal models of FASD, including aldehyde dehydrogenase (ALDH), Fancd2, Cdon, Gli2, Shh, Nos1, PDGFRA, hinfp, foxi1, mars, plk1 and vangl2; however, these genes are yet to be explored in human studies [201]. In humans, polymorphisms of alcohol dehydrogenase (ADH) genes have been the primary focus of investigation [199–201]. ADH enzymes oxidise ethanol to acetaldehyde and account for up to 95% of alcohol metabolism. ALDH and CYPE2E1 also influence ethanol metabolism in humans; however, their role in FASD susceptibility is yet to be established [200, 208]. Polymorphisms at the ADH1B locus produce enzymes that affect the rate of ethanol clearance. In populations of European ancestry, the slow-metabolising allele, ADH1B*1, is the typical variant and occurs in approximately 95% of individuals. The rare ADH1B*2 and ADH1B*3 alleles convert ethanol to acetaldehyde 75-88 times faster than ADH1B*1 [210]. Maternal and infant ADH1B genotype has been found to influence risk of FASD as, with the exception of one study [211], fast-metabolising alleles have been associated with a reduced risk of FASD symptomology following PAE [208, 212–215].

Lewis et al. explored the impact of ADH polymorphisms on children’s IQ following PAE [38]. Rare variants of four child single nucleotide polymorphisms (SNPs; ADH7 rs284779, ADH1B rs4147536, ADH1A rs975833 and ADH1A rs2866151; herein referred to as the SNP set) were negatively associated with IQ at age eight for children with low to moderate alcohol exposure in utero (1–6 units per week). These rare variants were hypothesised to be associated with slower metabolism, leading to relatively increased alcohol exposure and teratogenicity [38, 216]. However, a study of in vivo alcohol metabolism failed to show that these SNPs influenced blood or breath alcohol concentrations [217]. Within this study, another SNP, ADH4 rs4148884, had divergent effects. The rare allele was associated with decreased offspring IQ when present in the maternal genotype, but increased IQ when present in the child’s genotype. These SNPs were not associated with IQ among children of non-drinking mothers, suggesting that genotype acted as an effect modifier following maternal alcohol intake.

As well as influencing alcohol metabolism, genotype may influence drinking behaviour and, hence, the interest in ADH variants as instrumental variables in Mendelian randomisation studies [12]. Patterns of alcohol consumption tend to run in families and several genes have been found to contribute to risk of alcoholism [192, 196]. ADH1B has been widely studied and research shows that individuals with the rare allele consume less alcohol than those with the typical allele and have a reduced risk of alcoholism [218–221]. In pregnancy, mothers with the rare ADH1B allele have been found to consume significantly less alcohol before pregnancy and to be 50% less likely to binge drink during pregnancy [222]. Therefore, genotype may influence the risk of FASD through its influence on both alcohol metabolism and drinking behaviour [37].

### Using the DAG to support causal inferences and statistical analyses

**Fig. 2:**
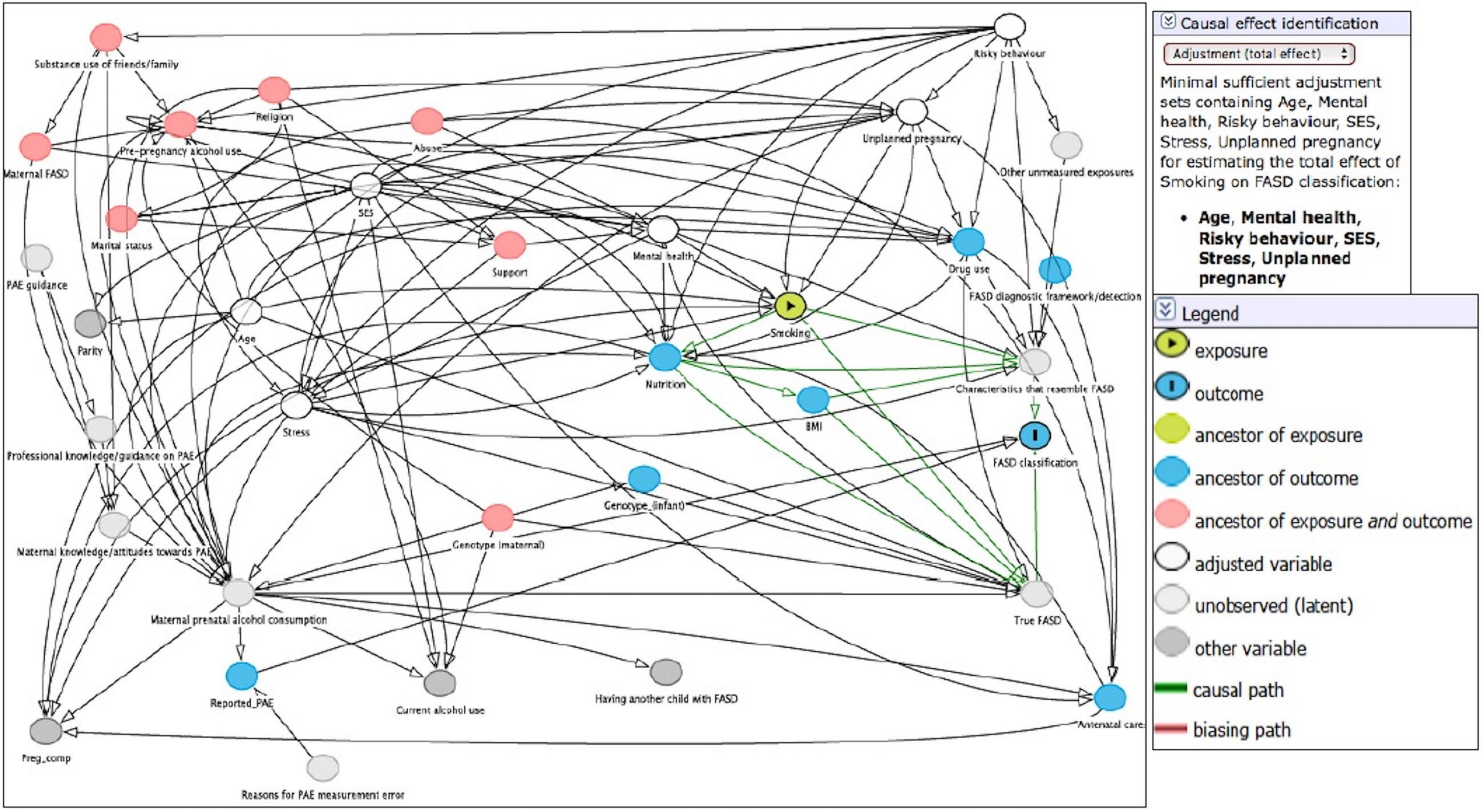
DAGitty output depicting which covariates should be included in the multivariable statistical model to minimise bias in the estimate of the total casual effect of prenatal smoking on FASD classification (the minimal sufficient adjustment set). Hypothesised causal pathways are depicted in green

To demonstrate how the DAG presented in this review can be used to clarify the causal pathways to FASD and to support statistical modelling strategies, we present a worked example using prenatal smoking as the risk factor of interest. We replicated the causal structure depicted in Fig. 1 in the DAGitty platform [27]. In all circumstances, there must have been prenatal alcohol exposure for there to be true FASD. The representation of the DAG that includes smoking as the key co-exposure in DAGitty is shown in Fig. 2.

There were six hypothesised causal pathways that accounted for the total causal effect of prenatal smoking on FASD classification among children with PAE.

These were

1. Prenatal smoking → ‘true’ FASD (unobserved) → FASD classification
2. Prenatal smoking → prenatal nutrition → ‘true’ FASD (unobserved) - > FASD classification
3. Prenatal smoking → prenatal nutrition → BMI → ‘true’ FASD (unobserved) → FASD classification
4. Prenatal smoking → characteristics that resemble FASD → FASD classification
5. Prenatal smoking → prenatal nutrition → characteristics that resemble FASD → FASD classification
6. Prenatal smoking → prenatal nutrition → BMI → characteristics that resemble FASD → FASD classification

Pathways 1–3 describe participants who have a pattern of symptoms that met the criteria for FASD and whose symptoms were caused by PAE. These are the participants with ‘true FASD.’ In contrast, pathways 4–6 describe participants who have characteristics that resemble FASD, but whose presentation has been caused by factors other than alcohol exposure. In practice, and in the absence of the full facial phenotype for FASD, it is often difficult to determine whether the characteristics that contribute to a diagnosis were caused by prenatal alcohol exposure or other factors and this requires expert clinical judgement. All these pathways contribute to the total prevalence of FASD that is observed. Based on the graphical rules described in Online Resource 1 and operationalised by DAGitty, if the postulated DAG is correct, with no omitted common causes of any two nodes and no measurement error (these are strong assumptions), then an unbiased estimate of the total causal effect of prenatal smoking on FASD could be obtained by adjusting for the following variables in the analysis: maternal age at pregnancy, prenatal mental health, maternal ‘risky behaviour’ (e.g. personality trait variable), SES, prenatal stress and unplanned pregnancy. It is important to bear in mind that DAGitty provides, if one exists, a *minimally sufficient adjustment set* (i.e. the minimum set of variables that is required to eliminate bias). In this smoking example, the minimally sufficient adjustment set suggested by the DAG was maternal age, mental health, risky behaviour, SES, stress and unplanned pregnancy. The researcher interested in pursuing this could enter these variables into a multivariable statistical model to minimise bias in the estimate of the total casual effect of prenatal smoking on FASD classification. If the number of events is low, then limiting to the minimal adjustment set may be a good idea. In practice, if the data allow further adjustment without running into numerical problems or issues of finite sample bias, then adjusting for additional predictors of the outcome is advisable, as this will lead to a more precise estimate which is still unbiased. It is important to check, however, that the inclusion of these additional variables does not induce bias. To check that adjustment for an additional variable is not going to induce bias, one can add candidate variables to the adjustment set in DAGitty to see if this creates biasing pathways between the exposure and the outcome. Online Resource 1 provides further explanation of when adjustment can lead to bias.

## Discussion

The aetiology of FASD is multifaceted and complex. The DAG that we have presented provides a summary of the distal and proximal factors that are proposed to causally influence FASD risk, as well as the factors that are proxies for causal factors or (non-causal) risk markers for FASD. Prior to pregnancy, multiple distal factors influence pregnancy planning and lifestyle choices including pre-pregnancy alcohol use. These factors are proposed to influence subsequent prenatal alcohol exposure. Following prenatal exposure to alcohol, FASD risk is determined by a range of lifestyle, sociodemographic, maternal, social, gestational and genetic factors that mutually influence children’s outcomes. The use of causal diagram theory as the underpinning framework for this evidence synthesis allows us to move beyond discussion of association to make inferences about potential causal relationships between factors relevant to FASD. The principles of causal diagram theory can be applied to this DAG to inform study design and statistical modelling strategies for FASD research.

## Strengths and limitations

### Literature review and DAG methodology

The literature review and DAG that we have presented build upon existing reviews of risk factors for FASD [17, 99, 223] and provide an updated synthesis using the latest evidence. The use of DAG methodology represents a novel approach to the study of FASD and adds clarity to causal inferences in an area that has mostly been confined to discussions of association. As a visual representation, the DAG presents a unified summary of the current evidence on the aetiology of FASD.

Since the DAG has been constructed based on current subject-matter knowledge and our interpretation of plausible causal structures, it may be subject to change as new evidence emerges. This is not a limitation of the DAG approach, but rather reflects the realities of making causal inferences within a developing evidence base. In its current form, the DAG that we have presented may be a useful working template for researchers of FASD, who can use this tool to guide their analyses and update the causal structure of the DAG, as necessary. DAGitty code is provided in Online Resource 3 and can be copied and pasted into the DAGitty platform to replicate the DAG that we present in this review. Users can then use DAGitty to inform their own modelling strategy, based on this DAG and/or update the DAG based on their own hypotheses and emerging evidence.

The DAG may also help researchers at the design stage, by indicating which variables are important to measure. It is important to note that the causal inferences derived from the DAG rest on the assumption that the diagram is valid. The DAG is based on our interpretation of the evidence and, therefore, it is possible that given the same evidence other researchers may depict the relationships differently. Furthermore, the limitations inherent in the evidence base will necessarily apply to the results from this review and the DAG. Many proposed risk factors for FASD, such as prenatal substance exposure and maternal stress, cannot be manipulated for ethical and practical reasons [224]. The evidence that we have used to construct the DAG was, therefore, based on experimental studies with animals and observational studies with humans. Animal studies have allowed the causal status of ethanol as a teratogen to be established [225] and have enabled causal conclusions about the influence of covariates such as stress and genetics on outcomes relevant to FASD [159, 207, 226]. Furthermore, in contrast to human studies, in which adverse exposures and health behaviours tend to co-occur, animal studies can isolate and evaluate the effects of specific exposures. However, animal models are imperfect substitutes for human studies for reasons including differences in alcohol metabolism, gestational processes, and their inability to replicate higher-level behavioural outcomes [7]. No single animal model has replicated all the attributes of FASD [225]. Observational studies of humans pose fewer concerns than animal studies in terms of exploring associations between a variety of co-occurring social and lifestyle factors and the full spectrum of FASD. However, this advantage is countered by the potential for bias due to the lack of experimental control. In general, observational studies are at risk of bias due to residual confounding, misclassification of the exposure and/or outcome, and differential loss to follow-up. In the evidence synthesis for the DAG, as well as studies with traditional observational designs, we included negative control and Mendelian randomisation studies. These studies are also subject to potential limitations. The contribution of paternal epigenetic factors to prenatal development in negative control studies remains to be established and effect estimates may be biased and imprecise in Mendelian randomisation studies if genetic instruments are weakly correlated with the exposure of interest and/or the instruments are invalid due to pleiotropy) [42, 221, 222, 227]. Nevertheless, the sources of bias differ for each approach and, under a triangulation method, the fact that the evidence from these different study designs points to a similar causal conclusion for many of the nodes of interest increases confidence in the causal hypotheses presented in the DAG [227, 228]. However, it may never be possible to determine whether the pathways suggested by the observational and animal studies represent the true causal pathways to FASD. Although this is a limitation of causal inference in general, compared to conventional statistical modelling strategies, approaches that make use of DAGs, when used sensibly, benefit from the increased transparency about causal assumptions and highlight likely remaining sources of bias.

#### The utility of DAGs in casual inference

Recently, there have been some criticisms about the gain in popularity of DAGs as tools to support causal inference. Kreiger and Davey Smith argue that DAGs have become synonymous with ‘casual inference’ and that this unnecessarily narrows the scope of causal inquiry in epidemiology [228]. They assert that “robust causal inference…comprises a complex narrative, created by scientists appraising, from diverse perspectives, different strands of evidence produced by myriad methods. DAGs can of course be useful, but should not alone wag the causal tale” [228] (p. 1789). We agree, and this is why we have drawn upon a broad body of evidence to support the causal pathways depicted in the DAG. Daniel et al. have countered that DAGs do not purport to provide a substitute for careful consideration of the evidence base. Instead they should be a result of this [229]. Under this view, we would contend that the narrative synthesis that we have conducted is an example of the *approach* encouraged by the causal inference ‘school’ of Pearl et al. [14, 230, 231], while the DAG is the *tool* that summarises key causal assumptions and enables application of graphical rules to guide appropriate analyses and interpretation.

Finally, DAGs are nonparametric and thus often attract criticism for not formally allowing the concept of effect modification to be visualised. This is particularly relevant when trying to describe the aetiological context of FASD. In this context, while there is a sole necessary cause (alcohol), several effect modifiers may be present that influence whether PAE results in FASD. If one views the DAG solely as a means of deciding on an adjustment set, then there is no need to depict effect modification. However, if (as we have done here), the DAG is also used as a visual summary of existing evidence in this area, then it is useful to enhance the DAG with a depiction of effect modification. In the DAG (Fig. 1), we have followed Weinberg [232] and Thompson’s [233] suggestion by representing effect modification with an arrow that intersects the intermediate causal pathway from PAE to FASD, although other representations have been suggested [234, 235]. The representation of effect modification as an intersecting arrow in the direct pathway between PAE and FASD is purely for visual illustration. To enable analysis in the DAGitty platform, we directly connected the arrows from the proposed effect modifiers to the FASD outcome to allow application of the graphical rule algorithms [236].

## Conclusions

The DAG presented in this review formalises and unifies current knowledge about the causal context of FASD. We believe that this DAG provides a useful synthesis of evidence for those interested in the aetiology of FASD and can be used as a tool to strengthen data collection and analysis strategies in studies of FASD risk factors.

## Electronic supplementary material

Below is the link to the electronic supplementary material.

Supplementary material 1 (DOCX 77 kb)
