# Supplementary material for: The causal web of foetal alcohol spectrum disorders: a review and causal diagram
Source: Eur Child Adolesc Psychiatry. 2019 Jan 16;29(5):575–94. doi: 10.1007/s00787-018-1264-3 (PMC7250957; doi:10.1007/s00787-018-1264-3)
Supplement: Supplementary file 1 — Supplementary material 1 (DOCX 77 kb) [file 787_2018_1264_MOESM1_ESM.docx]

Online resources

**The causal web of fetal alcohol spectrum disorders: a review and causal diagram**

**European Child & Adolescent Psychiatry**

**Authors:** McQuire C^1*^, Daniel R^2^, Hurt L^2^, Kemp A^2^, Paranjothy S^2^

^1^ Population Health Sciences, Bristol Medical School, University of Bristol, Canynge Hall, 39 Whatley Road, Bristol, BS8 2PS Email: [cheryl.mcquire@bristol.ac.uk](mailto:cheryl.mcquire@bristol.ac.uk)

**^2^** Division of Population Medicine, Cardiff University, 3rd Floor, Neuadd Meirionnydd, Heath Park, Cardiff, CF14 4YS

* Correspondence to: Dr Cheryl McQuire, Population Health Sciences, Bristol Medical School, University of Bristol, Canynge Hall, 39 Whatley Road, Bristol, BS8 2PS Email: cheryl.mcquire@bristol.ac.uk.

**Contents**

Online Resource 1: Causal diagram theory (directed acyclic graphs; DAGs)

Online Research 2: Search strings

Online Resource 3: DAGitty code (compatible with html version)

Online Resource 4: Categories of relationships implied by the literature review and DAG

Online Resource 1: Causal diagram theory (directed acyclic graphs; DAGs)

Counterfactuals and exchangeability

The counterfactual theory of causation has been used as a framework to support causal inference [1]. Under a counterfactual definition, causality refers to the notion “had the exposure differed, the outcome would have differed”[2]^(p. 1)^. Measures of association such as risk differences, risk ratios and odds ratios, which contrast the risk of an outcome between different groups of individuals, can be given a causal interpretation subject only to strong assumptions, the central of which is exchangeability. Exchangeability refers to the idea that the risk of the outcome in one exposure group (Group 1) would have been the same as the risk in another exposure group (Group 2), had the participants in Group 2 received the exposure given to Group 1, and vice-versa. Given exchangeability, the observed risk in Group 1 is equal to the counterfactual risk in both groups had everyone received the exposure given to Group 1, and the observed risk in Group 2 is equal to the counterfactual risk in both groups had everyone received the exposure given to Group 2.

In randomised controlled trials, the participants in different exposure groups are exchangeable, since treatment allocation is independent of participants’ pre-exposure characteristics. Measures of (conditional)^^[[1]](#footnote-1)^^ association from observational designs can also be used to estimate causal effects if conditional exchangeability is created by appropriate control of bias, such as adjustment for confounders [3]; that is, if exchangeability holds after conditioning on a set of observed variables. Causal diagrams can be used to help identify the optimal covariate set for adjustment.

Causal diagram language

Causal diagram theory presents its own language, which uses ancestry (family tree) terminology. This terminology is presented below, with an example based on Fig. S1, derived from the work of Greenland and colleagues [4].

*Fig. S1: Causal diagram example*


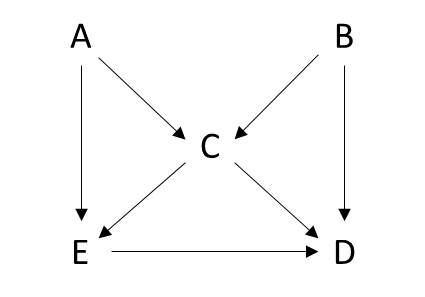


In Fig. S1, the variables depicted by letters (A, B, C, D, E) are called *nodes*. Arrows represent cause-effect relationships and are called *arcs* or *edges.* A *path* is any unbroken route that follows the arcs (regardless of direction) between adjacent nodes. *Causal* or *directed paths* are those which follow a sequence of arcs in a tail-to-head route, such as A → E → D. Any path which is not directed, is *undirected.* In particular, a path which starts with a head-to-tail arc is known as a *backdoor path*. In Fig. S1, all paths from E to D except E → D (e.g. E ← C → D) are backdoor paths.

A path is *blocked* at the point at which two arrowheads meet. The variable at which the arrowheads meet is called a *collider*. For example, in Fig. S1 the path A → C ← B → D is blocked by collider C. The arc from C to D represents a direct causal effect of C on D, as it is not intercepted by any of the other variables included in the diagram. In contrast, the causal path from C → E → D from C to D is indirect, as the effect is mediated by E. The absence of an arc, or any other open path, between A and B implies independence between A and B.

*Descendants* of a variable X are those that are affected directly or indirectly by X. For example, in Fig. S1, E, C and D are all descendants of A. More specifically, c*hildren* of a variable X are those that follow a single directed arc. In Fig. S1, D is a child of B, C and E. *Ancestors* or *causes* are variables that affect other variables directly or indirectly and, more specifically, *parents* are ancestor variables that are adjacent to the affected variable. In Fig. S1, A, B, C and E are all ancestors of D, and B, C and E are all parents of D.

Causal DAGs are directed, as the arcs connecting variables suggest a direction of effect, they are acyclic as they do not contain feedback loops^^[[2]](#footnote-2)^^ and they are causal as they include all common causes of a pair of variables [4-6].

Graphical properties and rules

Causal diagrams have several properties that are important to their interpretation. First, causal diagrams are qualitative and do not provide information about the strength or nature of association between two variables. For example, causal diagrams do not convey whether variables are categorical or continuous, whether dose-response relationships are linear or non-linear, whether causes are necessary or sufficient, whether there is effect modification, or whether effects are harmful or protective [4-6]. These properties are typically determined by statistical investigation of collected data.

Second, the *parent-child* and *direct/indirect* relationships implied by causal graphs are not inherent properties of the biologic relationship between two variables. This terminology simply reflects the level of detail that is captured in the diagram [4]. If we consider the relationship PAE → FASD, PAE is represented as a direct cause of FASD only because the specific intermediate causal mechanisms between PAE and FASD remain unknown and/or unmeasured or were simply not included in the DAG [7].

The presence of an arc between two nodes indicates a known or possible/probable causal relationship between two variables. If there is inconclusive evidence about whether there is a causal link between two variables, it is appropriate to include an arc between them (and in the direction deemed most plausible), since the presence of an arc indicates the *possibility* of an effect in the depicted direction whereas the absence of an arc or node indicates the stronger assumption of *no effect in either direction*, or at least the belief that the effect has a negligible impact given all other factors in the graph [8].

Finally, it is not necessary to include all causes of a variable within a causal diagram. However, if two variables share a common cause then this must be represented [5]. If this common cause is unmeasured then it must still be included and can be represented graphically as shown by node U in Fig. S2. Although not necessary, it is possible to include other variables that are not a common cause of two other variables [8], as in the case of node F in Fig. S2. For the purposes of the DAG developed in this review, we included some variables that are not common causes of two other variables to ensure complete coverage of the risk factors that have been described to date in the FASD literature. Sometimes, such variables are important in the analysis (e.g. to improve precision, to investigate effect modification/mediation), even if they are not important for reducing bias in the total causal effect estimate.

*Fig. S2: Causal diagram with unmeasured confounder (U), denoted by circle, and additional variable (F)*


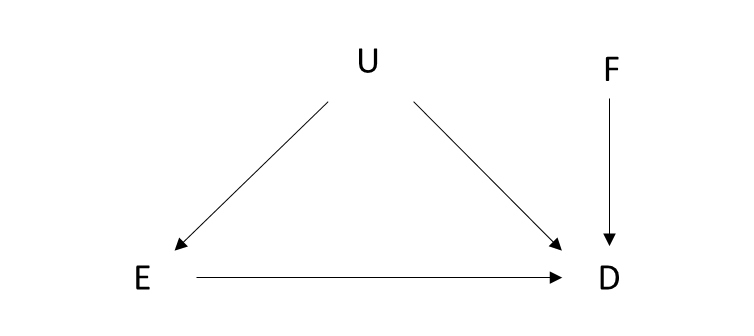


Two key concepts are integral to the interpretation and manipulation of DAGs. They are the *d-Separation* *criterion* and *Causal* *Markovian Condition* described by Pearl [9] and presented in an epidemiological context by Glymour and colleagues [5].

The d-Separation criterion is a set of graphical rules that can be used to infer independencies between variables. D-separation can be either unconditional or conditional. Unconditional d-separation occurs when there is no open path between two nodes. For example, in Fig. S3a, variables A and B are unconditionally d-separated, as the only paths between them are blocked by colliders C, D and E. Since variables A and B are d-separated, the graph predicts that, statistically, they will be marginally independent. In other words, causal diagram theory states that only open paths will create associations [5].

Conditioning has different implications for d-separation according to whether the node that is conditioned on is a collider or not [5,9]. Non-colliders may be mediating variables, as in Fig. S3b, where C is the mediator; or non-colliders may be a common cause of two variables as in Fig. S3c, where C is a confounder. Conditioning on a non-collider blocks the flow of statistical dependence along that path and (providing that there are no other open paths) creates d-separation and, therefore, statistical independence between A and D in Fig. S3b and E and D in Fig. S3c, conditional on C. Conversely, conditioning on a collider can *create* associations between two marginally independent variables. For example, in Fig. S3d the path between A and B is unconditionally blocked by collider C and so (in the absence of other paths from A to B) there is no marginal association between A and B; however, adjusting for C opens that path and creates a conditional association between A and B, given C. Fig. S3d provides a graphical representation of how an open path is created by conditioning on a collider. The dashed line in this Fig. is a non-directional arc that indicates that A and B are associated for reasons other than influencing each other or sharing a common cause, once C has been conditioned on (conditioning depicted by the square box) [4]. This dashed line is not formally a part of the DAG, but is often added informally to highlight the conditional associations that may be induced. Collider bias is explained more fully, with an intuitive example, in the next section.

A set of variables (S) is said to block the path between two nodes (e.g. E and D) if the path is closed after conditioning on this set. The set (S) unblocks the path if the path is open after conditioning. If there was no open path between E and D to begin with then the empty set separates them [5].

*Fig. S3: Causal diagrams for illustration of the d-separation criteria. Fig. a) presents the full diagram, where colliders C, E and D create unconditional separation between A and B. Fig. b) presents a causal pathway, where the causal effect between A and D is mediated by C, Fig. c) presents a biasing pathway between E and D due to not adjusting for confounder C and Fig. d) shows the introduction of a non-causal relationship between variables A and B (depicted by dashed line), due to inappropriate adjustment (depicted by square) on collider C.*

Bias

As well as facilitating expression of complex causal networks, DAGs have been advocated as useful tools for informing strategies for bias reduction [5,9]. Systematic bias is present when the chosen measure of association differs from the true causal effect [10]. Under these circumstances, different exposure groups differ in their probability of the outcome for reasons other than the effect of the exposure, even within strata of all the variables being conditioned upon in the analysis. This section will compare traditional and graphical approaches to defining and identifying confounding, selection, and information bias.

In epidemiology, confounding variables have commonly been defined by the following criteria [11]:

- The variable must be associated with both the exposure and the outcome.
- The variable must predict the outcome, independent of its association with the exposure.
- The variable must not lie on the causal pathway between the exposure and the outcome (i.e. it should not be a mediator).

Selection bias may also lead to misleading effect estimates within epidemiological studies. Selection bias occurs when the study population does not represent the target population [12] or, more formally, when:

“the association between exposure and disease includes a non-causal component attributable to restricting the analysis to certain level(s) of a common effect of exposure and disease or, more generally, to conditioning on a common effect of variables correlated with exposure and disease”[13]^(p. 182)^.

In contrast to traditional epidemiological approaches, graphical methods offer a more precise definition by expressing bias as any unblocked backdoor path between the exposure and outcome. For example, in Fig. S3a, all paths from E to D, except for the direct path from E → D are backdoor paths. Therefore, the unadjusted estimate for the effect of E on D will be partly due to the causal effect and partly due to the remaining biasing paths [4]. Some argue that because graphical rules are sufficient to identify structural sources of bias there is no need to distinguish between confounding and selection bias. Nevertheless, for the purposes of unifying graphical and epidemiological definitions of bias it is useful to indicate how these concepts overlap [5]. Also, knowing whether the source of bias is due to confounding or selection issues may suggest approaches for minimising bias by study design.

Graphical methods depict confounding as an open backdoor path formed by a common cause of two variables. In many instances, application of the traditional and graphical criteria for confounding identify the same variables as confounders and would recommend similar strategies for control of this bias.

*Fig. S4: Partial a) and full version b) of a causal diagram to illustrate complexities when adjusting for confounders, including over-adjustment when C is both a confounder and collider c).*

For example, Fig. S4a is a sub-section of Fig. S4b and demonstrates that the effect estimate for the relationship between E and D is partly confounded by C. If we take Fig. S4a to be a complete DAG (i.e. assume that there are no further common causes that are not represented in the graph), then the traditional and graphical approaches would both identify C as a confounder and would both suggest C as a covariate that should be controlled for to gain an unbiased estimate of the effect of E on D. However, the traditional and graphical approaches to confounding sometimes diverge. Greenland and colleagues [4] presented the full DAG (Fig. S4b) to colleagues and asked them identify the smallest subset of variables that would be sufficient to adjust for in order to create an unbiased estimate of the effect of E on D. Most suggested that adjusting for A or B only would not be sufficient, but that adjusting for C alone would be sufficient. This choice was based on the reasoning that adjustment for only A and B would not resolve the confounding by C. Adjustment for C, however, would block the pathway between A and D, given E, and would leave B unassociated with E. Therefore, A and B would no longer meet the traditional criteria for confounding, as they would not be associated with both the exposure and the outcome. However, the graphical criteria for confounding indicate that controlling for C alone would not be sufficient to eliminate bias. This is because C is a collider (a common effect of A and B, on the pathway A → C ← B). As previously described, conditioning on a collider creates an association between its parents. This, in turn, creates a new backdoor pathway, which, if uncontrolled, will lead to a biased effect estimate between E and D (see Fig. S4c). Therefore, to obtain an unbiased estimate of E on D, it is also necessary to control for A or B, in addition to C [4].

Collider bias arises because observing information about one of the common causes of an effect, makes the other cause more or less likely given the occurrence of that effect, even if these causes were previously independent [9]. Hernan provides the following example [13]. Suppose that dieting and a particular form of cancer are statistically independent and, therefore, knowing that someone was on a diet does not change your knowledge about their risk of cancer. A common effect of dieting and cancer is weight loss. Given that we know that someone had lost weight (i.e. we condition on weight loss), cancer and diet no longer remain independent. This is because knowing that someone had lost weight and was not on a diet increases the probability that this person has cancer (intuitively: if they lost weight but weren’t on a diet then it must have been something else [e.g. cancer] that caused them to lose weight). Therefore, within categories of weight loss, dieting and cancer become inversely associated. In these circumstances the crude effect estimate of the relationship between diet and cancer is unbiased and conditioning on collider weight loss creates a spurious association between diet and cancer, thus leading to a biased adjusted estimate.

Collider bias is a form of selection bias. Selection bias due to loss to follow-up and missing data can also be represented graphically [14]. These forms of bias occur when study drop-out or completeness of data are associated with both the exposure and the outcome [11]. Restriction of analysis to participants with complete data represents a form of conditioning on the common effect of exposure and outcome - data availability.

In summary, DAGs can assist with the identification and representation of bias as well as assisting with the choice of adjustment variables that will help to remove or reduce that bias. Graphical methods can be applied to complex causal networks in situations where traditional criteria for confounder identification may fail [4]. Bias due to confounding can typically be reduced via multivariable regression modelling, using the method outlined above to identify suitable covariates. Selection bias can be minimised by avoiding harmful adjustment on common effects of the exposure and outcome, or by using appropriate methods to account for missing data.

Online Resource 2: Search strings

**Search 1a: Medline search for review articles on FASD risk factors**

1. Fetal alcohol spectrum disorders/

2. Risk factors/

3. 1 and 2

4. f?etal alcohol syndrome.tw.

5. FASD.tw.

6. risk factor*.tw.

7. 4 or 5

8. 6 and 7

9. 3 or 8

10. systematic review.mp.

11. 9 and 10

**Search 1b: Medline search for articles on FASD and stress**

1. Fetal alcohol spectrum disorders/

2. f?etal alcohol syndrome.tw.

3. FASD.tw.

4. STRESS, PSYCHOLOGICAL/ or stress.mp.

5. 1 and 4

6. 2 or 3

7. 4 and 6

8. 5 or 7

9. alcohol*.mp.

10. 4 and 9

11. Pregnancy/

12. pregnan*.mp.

13. 11 or 12

14. 10 and 13

15. 8 or 14

Online Resource 3: DAGitty code (compatible with html version)

dag {

"Antenatal care" [pos="0.945,1.014"]

"Characteristics that resemble FASD" [latent,pos="0.880,0.596"]

"Current alcohol use" [pos="0.367,0.996"]

"Drug use" [pos="0.823,0.446"]

"FASD classification" [outcome,pos="0.880,0.688"]

"FASD diagnostic framework/detection" [pos="0.885,0.480"]

"Genotype (maternal)" [pos="0.417,0.791"]

"Genotype_(infant)" [pos="0.543,0.742"]

"Having another child with FASD" [pos="0.562,0.982"]

"Marital status" [pos="0.092,0.417"]

"Maternal FASD" [pos="0.018,0.327"]

"Maternal knowledge/attitudes towards PAE" [latent,pos="0.086,0.800"]

"Maternal prenatal alcohol consumption" [latent,pos="0.193,0.883"]

"Mental health" [pos="0.559,0.430"]

"Other unmeasured exposures" [latent,pos="0.956,0.326"]

"PAE guidance" [latent,pos="0.019,0.466"]

"Pre-pregnancy alcohol use" [pos="0.143,0.299"]

"Professional knowledge/guidance on PAE" [latent,pos="0.074,0.680"]

"Reasons for PAE measurement error" [latent,pos="0.266,1.102"]

"Risky behaviour" [pos="0.816,0.210"]

"Substance use of friends/family" [pos="0.079,0.190"]

"True FASD" [latent,pos="0.882,0.884"]

"Unplanned pregnancy" [pos="0.774,0.283"]

Abuse [pos="0.356,0.295"]

Age [pos="0.200,0.533"]

BMI [pos="0.689,0.643"]

Nutrition [pos="0.561,0.590"]

Parity [pos="0.065,0.548"]

Preg_comp [pos="0.027,1.056"]

Religion [pos="0.224,0.256"]

Reported_PAE [pos="0.196,0.987"]

SES [pos="0.303,0.376"]

Smoking [pos="0.669,0.526"]

Stress [pos="0.243,0.650"]

Support [pos="0.427,0.450"]

"Antenatal care" -> Nutrition [pos="0.785,0.551"]

"Antenatal care" -> Preg_comp [pos="0.905,1.106"]

"Characteristics that resemble FASD" -> "FASD classification"

"Drug use" -> "Antenatal care" [pos="0.966,0.558"]

"Drug use" -> "Characteristics that resemble FASD"

"Drug use" -> "True FASD" [pos="0.803,0.689"]

"Drug use" -> Nutrition [pos="0.788,0.580"]

"Drug use" -> Preg_comp [pos="-0.028,0.208"]

"FASD diagnostic framework/detection" -> "Characteristics that resemble FASD"

"Genotype (maternal)" -> "Current alcohol use"

"Genotype (maternal)" -> "Genotype_(infant)"

"Genotype (maternal)" -> "Maternal prenatal alcohol consumption"

"Genotype (maternal)" -> "Pre-pregnancy alcohol use" [pos="0.004,0.425"]

"Genotype (maternal)" -> "True FASD"

"Genotype_(infant)" -> "True FASD"

"Marital status" -> "Unplanned pregnancy"

"Marital status" -> SES

"Marital status" -> Support

"Maternal FASD" -> "Maternal prenatal alcohol consumption" [pos="-0.030,0.659"]

"Maternal FASD" -> "Mental health"

"Maternal FASD" -> "Pre-pregnancy alcohol use"

"Maternal knowledge/attitudes towards PAE" -> "Maternal prenatal alcohol consumption"

"Maternal prenatal alcohol consumption" -> "Antenatal care" [pos="0.597,0.921"]

"Maternal prenatal alcohol consumption" -> "Current alcohol use"

"Maternal prenatal alcohol consumption" -> "FASD classification"

"Maternal prenatal alcohol consumption" -> "Having another child with FASD" [pos="0.450,0.927"]

"Maternal prenatal alcohol consumption" -> "True FASD"

"Maternal prenatal alcohol consumption" -> Nutrition [pos="0.035,0.530"]

"Maternal prenatal alcohol consumption" -> Preg_comp

"Maternal prenatal alcohol consumption" -> Reported_PAE

"Mental health" -> "Characteristics that resemble FASD"

"Mental health" -> "Drug use"

"Mental health" -> "Maternal prenatal alcohol consumption" [pos="-0.011,0.114"]

"Mental health" -> "True FASD" [pos="0.585,0.686"]

"Mental health" -> Nutrition

"Mental health" -> Smoking

"Mental health" -> Stress

"Other unmeasured exposures" -> "Characteristics that resemble FASD"

"PAE guidance" -> "Maternal knowledge/attitudes towards PAE"

"PAE guidance" -> "Professional knowledge/guidance on PAE"

"Pre-pregnancy alcohol use" -> "Current alcohol use" [pos="0.400,0.812"]

"Pre-pregnancy alcohol use" -> "Maternal prenatal alcohol consumption" [pos="-0.023,0.339"]

"Pre-pregnancy alcohol use" -> "Unplanned pregnancy" [pos="0.519,0.362"]

"Pre-pregnancy alcohol use" -> Nutrition [pos="0.569,0.369"]

"Professional knowledge/guidance on PAE" -> "Maternal knowledge/attitudes towards PAE"

"Reasons for PAE measurement error" -> Reported_PAE

"Risky behaviour" -> "Characteristics that resemble FASD"

"Risky behaviour" -> "Drug use"

"Risky behaviour" -> "Maternal prenatal alcohol consumption" [pos="-0.016,0.266"]

"Risky behaviour" -> "Other unmeasured exposures"

"Risky behaviour" -> "Pre-pregnancy alcohol use" [pos="0.333,0.249"]

"Risky behaviour" -> "Substance use of friends/family"

"Risky behaviour" -> "Unplanned pregnancy"

"Risky behaviour" -> Nutrition [pos="0.613,0.221"]

"Risky behaviour" -> Smoking [pos="0.640,0.289"]

"Risky behaviour" -> Stress [pos="0.423,0.260"]

"Substance use of friends/family" -> "Maternal FASD"

"Substance use of friends/family" -> "Maternal knowledge/attitudes towards PAE"

"Substance use of friends/family" -> "Maternal prenatal alcohol consumption" [pos="-0.011,0.474"]

"Substance use of friends/family" -> "Pre-pregnancy alcohol use"

"True FASD" -> "FASD classification"

"Unplanned pregnancy" -> "Antenatal care" [pos="0.987,0.439"]

"Unplanned pregnancy" -> "Drug use"

"Unplanned pregnancy" -> "Maternal prenatal alcohol consumption" [pos="0.442,0.381"]

"Unplanned pregnancy" -> Nutrition [pos="0.785,0.560"]

"Unplanned pregnancy" -> Smoking

Abuse -> "Antenatal care" [pos="0.975,0.247"]

Abuse -> "Mental health"

Abuse -> "Unplanned pregnancy" [pos="0.562,0.254"]

Abuse -> Stress [pos="0.010,0.344"]

Age -> "Drug use" [pos="0.211,0.344"]

Age -> "Maternal prenatal alcohol consumption" [pos="-0.007,0.629"]

Age -> "Pre-pregnancy alcohol use"

Age -> "True FASD"

Age -> Parity [pos="0.104,0.543"]

Age -> Smoking [pos="0.359,0.474"]

BMI -> "Characteristics that resemble FASD"

BMI -> "True FASD"

Nutrition -> "Characteristics that resemble FASD" [pos="0.662,0.618"]

Nutrition -> "True FASD" [pos="0.626,0.703"]

Nutrition -> BMI

Nutrition -> Preg_comp [pos="0.038,0.380"]

Religion -> "Current alcohol use"

Religion -> "Marital status" [pos="0.249,0.354"]

Religion -> "Maternal prenatal alcohol consumption" [pos="-0.031,0.231"]

Religion -> "Pre-pregnancy alcohol use"

Religion -> "Unplanned pregnancy" [pos="0.551,0.230"]

Religion -> Support

Reported_PAE -> "FASD classification"

SES -> "Antenatal care" [pos="0.626,1.067"]

SES -> "Current alcohol use"

SES -> "Drug use" [pos="0.337,0.267"]

SES -> "Maternal prenatal alcohol consumption" [pos="-0.021,0.498"]

SES -> "Mental health"

SES -> "Pre-pregnancy alcohol use" [pos="-0.008,0.260"]

SES -> "Unplanned pregnancy" [pos="0.376,0.309"]

SES -> Nutrition [pos="0.467,0.366"]

SES -> Parity [pos="0.084,0.458"]

SES -> Smoking [pos="0.432,0.349"]

SES -> Stress

SES -> Support

Smoking -> "Characteristics that resemble FASD"

Smoking -> "True FASD"

Smoking -> Nutrition

Smoking -> Preg_comp [pos="0.036,0.523"]

Stress -> "Characteristics that resemble FASD" [pos="0.548,0.761"]

Stress -> "Drug use" [pos="0.292,0.114"]

Stress -> "Maternal prenatal alcohol consumption" [pos="0.165,0.799"]

Stress -> "True FASD"

Stress -> Nutrition [pos="0.429,0.691"]

Stress -> Preg_comp [pos="0.085,0.802"]

Stress -> Smoking

Support -> "Mental health"

Support -> Stress

}

Online Resource 4: Categories of relationships implied by the literature review and DAG

Causal risk factors

Given that prenatal alcohol exposure is the sole necessary cause for FASD, other causal risk factors were hypothesised to influence the risk of FASD in two ways: by changing the risk of alcohol exposure during pregnancy and/or by changing the effect of alcohol on the fetus. These causal risk factors are described below.

Prenatal alcohol use is the sole necessary cause of FASD and thus is the *primary causal risk factor*. The effect of maternal alcohol use is mediated by proximal unmeasured causal processes, or provocative factors, that include fetal alcohol exposure, hypoxia, free radical damage, epigenetic changes and disrupted neurotransmitter functioning [7].

Although PAE is the sole necessary cause of FASD, it is also useful to study exposures that modify the effect of PAE on FASD to identify opportunities for harm reduction. These are known as *effect modifiers*. Identified effect modifiers include maternal age, genotype, prenatal stress, prenatal mental health, prenatal nutrition, BMI, prenatal smoking, and prenatal illicit drug use [15-17]. Within this group of effect modifiers, some are potentially amenable to intervention (prenatal mental health, prenatal stress, prenatal nutrition, BMI, prenatal smoking, and prenatal illicit drug use) and some are not (maternal age, genotype). Modelling strategies that estimate the effect of proposed causal effect modifiers on FASD can suggest useful potential targets for intervention.

Most of the risk factors that have been identified for FASD feature at a more distal stage in the causal pathway. We refer to these as *distal causal risk factors*. Distal factors include: substance use of friends and family, social support, socioeconomic status, marital status, maternal FASD symptomology, alcohol use before pregnancy, religion, abuse and unplanned pregnancy. Although these factors feature at a more distal stage of the causal pathway to FASD, some may be useful targets for intervention. For example, if low social support increases the risk of FASD by increasing prenatal stress, which in turn increases the risk of PAE, then social support initiatives may be a useful focus for intervention.

Non-causal factors (risk markers)

Having another child with FASD and postnatal alcohol use are depicted as *risk markers* for FASD. These factors do not lie on the causal pathway between substance use in the current pregnancy and FASD, since they occur outside of the index pregnancy. Nevertheless, they may be useful indicators of adverse prenatal exposures and, therefore, may be informative for identifying children who require follow-up and assessment.

Other categories

In the preceding sections, we described categories of causal risk factors and risk markers for FASD. Here, we specify other nodes that were included in the DAG to represent potential sources of bias and measurement error.

First, adverse exposures and events such as substance use, unhealthy diet, life stressors and adverse pre- and postnatal environments, tend to co-occur [20-22]. Rather than one of these factors simply causing the others, it is likely that their co-occurrence reflects some latent common cause. This common cause has been labelled ‘risky behaviour’ in some DAGs that link alcohol use and smoking, and in studies that explore binge drinking and unplanned pregnancy [8,23]. Therefore, for the DAG that we present in this review, we have included an overarching node labelled “*risky behaviour/unspecified common causes of maternal alcohol use and other adverse exposures”* to represent the latent factors that cause adverse exposures to co-occur. The clustering of adverse exposures may be due to a variety of unknown and unmeasured factors. Therefore, this common cause node was added to the DAG to represent this potential source of residual confounding.

Second, in many epidemiological studies, it is not possible to directly measure the true exposure and true outcome. Therefore, it is necessary to rely on surrogate measures that approximate but do not perfectly match the values of the true exposure and outcome [24]. The degree of bias depends on the extent to which the measured values deviate from the true values [25]. Measurement error of fetal alcohol exposure and of FASD is a particular concern. Fetal alcohol exposure is influenced not only by maternal alcohol consumption, but also by maternal and fetal metabolism, and a range of other factors that modify the teratogenic effects of alcohol [26]. Often, only one of these components - maternal alcohol consumption – is observed, with a degree of measurement error. Ascertainment of maternal alcohol use normally relies on maternal self-report or incomplete medical records, which are thought to underestimate true maternal alcohol use [27,28]. Therefore, in the absence of reliable measures of fetal blood alcohol concentration, there will be measurement error of true PAE. Therefore, in the DAG, we depicted self-reported maternal alcohol use as a source of measurement error. It is a factor that is associated with, but distinct from, true maternal alcohol use.

Measurement error of the primary exposure (true maternal alcohol use) will also contribute to measurement error of the outcome (FASD diagnosis), since alcohol use is a diagnostic criterion for FASD. Therefore, for reasons of transparency, we represented these sources of error as extra nodes in the DAG. It is important to note that measurement error is implicitly acknowledged for all reported exposures in the DAG (e.g. prenatal smoking, prenatal stressful life events), however we chose not to depict these factors as extra nodes for reasons of visual clarity.

FASD diagnosis is a surrogate for true FASD status. FASD diagnosis is influenced by a range of factors. These factors are represented as separate nodes within the DAG and include: the choice of diagnostic framework, adequacy of FASD detection (e.g. the availability of diagnostic services for FASD), and consideration of the role of other exposures that can lead to symptoms that resemble FASD (for example the effect of prenatal tobacco smoking on low birth weight) [29]. The FASD Canadian guidelines for diagnosis (2005), describe the challenge of diagnosis as follows: “The face of FAS is the result of a specific effect of ethanol teratogenesis altering growth of the midface and brain. Those exposed to other embryotoxic agents may display a similar, but not identical, phenotypic facial development, impaired growth, a higher frequency of anomalies and developmental and behavioural abnormalities... Knowledge of exposure history will decrease the possibility of misdiagnosing FASD”[30]^(pS7)^. As FASD shares many overlapping features with other disorders, it has been described as a diagnosis of exclusion [31].

The final node in this category was introduced to depict *biological mechanisms (provocative factors)*. The specific biological mechanisms that cause FASD are still under investigation [7]. Therefore, within the DAG, we depicted these provocative factors as a broad category of unmeasured factors that mediate the relationship between PAE and FASD (denoted as a large connecting arrow between true prenatal alcohol use and true FASD).

References

1. Maldonado G, Greenland S (2002) Estimating causal effects. Int J Epidemiol 31:422-429. doi:10.1093/ije/31.2.422

2. Shrier I, Platt RW (2008) Reducing bias through directed acyclic graphs. BMC Med Res Methodol 8:70. http://dx.doi.org/10.1186/1471-2288-8-70.

3. Kramer M (2015) Uses and misuses of causal language. BJOG 122:462-463

4. Greenland S, Pearl J, Robins JM (1999) Causal diagrams for epidemiologic research. Epidemiology 10:37-48

5. Glymour MM, Greenland S (2008) Causal Diagrams. In: Rothman KJ, Greenland S, Lash, TL (ed) Modern epidemiology. Lippincott Williams & Wilkins, Philadelphia, pp 183-209

6. Hernán MA, Robins JM (2016) Causal Inference. Chapman & Hall/CRC, Boca Raton, forthcoming; http://www.hsph.harvard.edu/miguel-hernan/causal-inference-book/. Accessed February 10, 2016.

7. British Medical Association (BMA) (2016) Alcohol and pregnancy: preventing and managing fetal alcohol spectrum disorders. BMA: London. https://www.bma.org.uk/collective-voice/policy-and-research/public-and-population-health/alcohol/alcohol-and-pregnancy. Accessed 23 May 2017

8. Daniel R (2015) An introduction to causal inference: counterfactuals and causal diagrams. In: Advanced Course in Epidemiological Analysis, London School of Hygiene and Tropical Medicine, London

9. Pearl J (2009) Causality: models, reasoning and inference, 2nd edn. Cambridge University Press, New York

10. Hernan M (2004) A definition of causal effect for epidemiological research. J Epidemiol and Commun Health 58:265-271

11. Hennekens CH, Buring JE, Mayrent SL (1987) Epidemiology in medicine. Little Brown and Company, Boston

12. Delgado-Rodríguez M, Llorca J (2004) Bias. J Epidemiol Community Health 58:635-641

13. Hernán MA, Hernández-Díaz S, Werler MM, Mitchell AA (2002) Causal knowledge as a prerequisite for confounding evaluation: an application to birth defects epidemiology. Am J Epidemiol 155:176-184

14. Hernán MA, Hernández-Díaz S, Robins JM (2004) A structural approach to selection bias. Epidemiology 15:615-625

15. Esper LH, Furtado EF (2014) Identifying maternal risk factors associated with fetal alcohol spectrum disorders: a systematic review. Eur Child Adolesc Psychiatry 23:877-889. doi:10.1007/s00787-014-0603-2

16. Young JK, Giesbrecht HE, Eskin MN, Aliani M, Suh M (2014) Nutrition implications for fetal alcohol spectrum disorders. Adv Nutr 5:675-692

17. Norberg Å, Jones AW, Hahn RG, Gabrielsson JL (2003) Role of variability in explaining ethanol pharmacokinetics. Clin Pharmacokinet 42:1-31

18. Janisse JJ, Bailey BA, Ager J, Sokol RJ (2014) Alcohol, tobacco, cocaine, and marijuana use: relative contributions to preterm delivery and fetal growth restriction. Subst Abus 35:60-67. doi:10.1080/08897077.2013.804483

19. Lange S, Probst C, Quere M, Rehm J, Popova S (2015) Alcohol use, smoking and their co-occurrence during pregnancy among Canadian women, 2003 to 2011/12. Addict Behav 50:102-109.

20. Keegan J, Parva M, Finnegan M, Gerson A, Belden M (2010) Addiction in pregnancy. J Addict Dis 29:175-191

21. Schempf AH, Strobino DM (2008) Illicit drug use and adverse birth outcomes: is it drugs or context? J Urban Health 85:858-873. doi:10.1007/s11524-008-9315-6

22. Moller M, Karaskov T, Koren G (2010) Opioid detection in maternal and neonatal hair and meconium: characterization of an at-risk population and implications to fetal toxicology. Ther Drug Monit 32:318-323

23. Naimi TS, Lipscomb LE, Brewer RD, Gilbert BC (2003) Binge drinking in the preconception period and the risk of unintended pregnancy: implications for women and their children. Pediatrics 111:1136-1141

24. Shahar E (2009) Causal diagrams for encoding and evaluation of information bias. J Eval Clin Pract 15:436-440

25. Hernán MA, Cole SR (2009) Invited commentary: causal diagrams and measurement bias. Am J Epidemiol 170: 959-62

26. Scholder S, Wehby GL, Lewis S, Zuccolo L (2014) Alcohol exposure in utero and child academic achievement. Econ J 124:634-667. http://dx.doi.org/10.1111/ecoj.12144

27. Ernhart CB, Morrow‐Tlucak M, Sokol RJ, Martier S (1988) Underreporting of alcohol use in pregnancy. Alcohol Clin Exp Res 12:506-511

28. Feunekes GI, van't Veer P, van Staveren WA, Kok FJ (1999) Alcohol intake assessment: the sober facts. Am J Epidemiol 150:105-112

29. Chasnoff IJ, Wells AM, King L (2015) Misdiagnosis and missed diagnoses in foster and adopted children with prenatal alcohol exposure. Pediatrics 135: 264-270. doi:10.1542/peds.2014-2171

30. Chudley AE, Conry J, Cook JL, Loock C, Rosales T, LeBlanc N (2005) Fetal alcohol spectrum disorder: Canadian guidelines for diagnosis. CMAJ 172:S1-S21. https://doi.org/10.1503/cmaj.1040302

31. Mukherjee RAS (2013) Fetal alcohol spectrum disorders: diagnosis and complexities. In: Carpenter B, Blackburn C, Egerton J (eds) Fetal alcohol spectrum disorders: interdisciplinary perspectives. Routledge, London, pp 159-173.

1. Conditional associations refer to adjusted effect estimates. Marginal associations are unadjusted (crude) effect estimates. [↑](#footnote-ref-1)
2. The absence of feedback loops means that a variable cannot be an ancestor or descendant of itself such that X causes Y and Y simultaneously causes X. If the value of X affects Y and then Y affects a later value of X this temporal sequence can be represented in separate variables (e.g. X_0_ → Y_0_ → X_1_). This coincides with our intuition on causality: nothing is its own cause. [↑](#footnote-ref-2)
